# Altered Gap Junction Network Topography in Mouse Models for Human Hereditary Deafness

**DOI:** 10.3390/ijms21197376

**Published:** 2020-10-06

**Authors:** Sara Eitelmann, Laura Petersilie, Christine R. Rose, Jonathan Stephan

**Affiliations:** 1 Animal Physiology Group, Department of Biology, University of Kaiserslautern, Erwin Schrödinger-Straße 13, D 67663 Kaiserslautern, Germany; sara.eitelmann@uni-duesseldorf.de; 2 Institute of Neurobiology, Heinrich Heine University Düsseldorf, Universitätsstraße 1, D 40225 Düsseldorf, Germany; laura.petersilie@hhu.de (L.P.); rose@uni-duesseldorf.de (C.R.R.)

**Keywords:** astrocytes, auditory brainstem, lateral superior olive, gap junctions, voltage-activated calcium channel 1.3, otoferlin, spontaneous activity, deafness

## Abstract

Anisotropic gap junctional coupling is a distinct feature of astrocytes in many brain regions. In the lateral superior olive (LSO), astrocytic networks are anisotropic and oriented orthogonally to the tonotopic axis. In Ca_V_1.3 knock-out (KO) and otoferlin KO mice, where auditory brainstem nuclei are deprived from spontaneous cochlea-driven neuronal activity, neuronal circuitry is disturbed. So far it was unknown if this disturbance is also accompanied by an impaired topography of LSO astrocyte networks. To answer this question, we immunohistochemically analyzed the expression of astrocytic connexin (Cx) 43 and Cx30 in auditory brainstem nuclei. Furthermore, we loaded LSO astrocytes with the gap junction-permeable tracer neurobiotin and assessed the network shape and orientation. We found a strong elevation of Cx30 immunoreactivity in the LSO of Ca_V_1.3 KO mice, while Cx43 levels were only slightly increased. In otoferlin KO mice, LSO showed a slight increase in Cx43 as well, whereas Cx30 levels were unchanged. The total number of tracer-coupled cells was unaltered and most networks were anisotropic in both KO strains. In contrast to the WTs, however, LSO networks were predominantly oriented parallel to the tonotopic axis and not orthogonal to it. Taken together, our data demonstrate that spontaneous cochlea-driven neuronal activity is not required per se for the formation of anisotropic LSO astrocyte networks. However, neuronal activity is required to establish the proper orientation of networks. Proper formation of LSO astrocyte networks thus necessitates neuronal input from the periphery, indicating a critical role of neuron-glia interaction during early postnatal development in the auditory brainstem.

## 1. Introduction

In many brain regions, astrocytes and oligodendrocytes form large panglial gap junction (GJ)-mediated networks [1,2,3,4]. In the hippocampus, where only few oligodendrocytes are located [1,5], networks mainly consist of astrocytes [6]. GJ networks exhibit a heterogeneous topography throughout the CNS. In particular areas, astrocytes are unequally interconnected to each other leading to an anisotropic network topography. Such limitations are present, for example, in sensory systems, which exhibit a strong anatomo-functional organization. In the barrel cortex and the barreloid thalamus, tracer coupling is restricted to the barrels [7,8]. Moreover, anisotropic tracer spread is present in the lateral superior olive (LSO) and the inferior colliculus (IC) [1,2,9]—two nuclei of the auditory brainstem, in which tracer-coupled networks are oriented orthogonally to the tonotopic axis. Both LSO and IC are tonotopically organized [10,11,12] and principal neurons refer to this organization, as their dendritic trees exhibit a narrow morphology with an orientation orthogonal to the tonotopic axis [13,14,15,16,17]. The correlation of astrocyte network anisotropy with the topography of principal neurons suggests that they are causally linked to each other, though the mechanism is still unknown.

Before hearing onset, which takes place at around postnatal day 12 in mice, circuits undergo substantial refinement [10,18,19]. In the superior olivary complex (SOC) of some species, namely rats and gerbils, but not in mice, principal neurons in the medial nucleus of the trapezoid (MNTB) change their synaptic phenotype from GABAergic towards glycinergic [20,21,22]. Furthermore, the number of MNTB–LSO projections decreases within the first two postnatal weeks, and MNTB–LSO synapses become consolidated [22,23]. This developmental refinement requires spontaneous cochlea-driven neuronal activity [24,25,26]. Even interfering with cholinergic efferent signaling onto hair cells in the cochlea alters spontaneous cochlea-driven neuronal activity and causes disturbed tonotopic map formation and impairment of sound source localization [27,28]. Mutations in various genes, for example coding for the voltage-activated calcium channel (Ca_V_) 1.3 or the calcium sensor otoferlin in inner hair cells of the cochlea, cause hereditary deafness [29,30,31,32]. For both deafness genes, mouse models are available: Ca_V_1.3 knock-out (KO) mice [33] and otoferlin KO mice [34]. In these mice, the auditory brainstem lacks spontaneous cochlea-driven neuronal activity, which in the SOC results in malformed nuclei and impaired circuit formation, i.e., reduced refinement and strengthening of MNTB–LSO synapses as well as impaired reorganization of the dendrite topography of LSO principal neurons [26,35,36]. In the wild type (WT), LSO astrocyte networks are anisotropic and predominantly oriented orthogonally to the tonotopic axis, thus correlating with dendrite topography and tonotopy. It has been suggested that network anisotropy might be beneficial for directed redistribution of, e.g., ions to limit crosstalk between neighboring isofrequency bands [1]. Accordingly, any impairment of network anisotropy and preferential orientation would further undermine tonotopic information processing. However, it was unknown so far if astrocytes and astrocytic networks are affected in the two KOs models.

Our results show that LSO astrocytes assessed at postnatal days 10–12 maintain an electrophysiologically earlier developmental phenotype in Ca_V_1.3 KO and otoferlin KO mice. The expression of connexin (Cx) 43 and Cx30 was increased, but the degree of GJ coupling was unaltered. However, network topography was strongly altered in Ca_V_1.3 KO and otoferlin KO mice. Most networks were anisotropic, but in contrast to the WT, networks were now predominantly oriented parallel (and not orthogonal) to the tonotopic axis. Thus, our data show that spontaneous cochlea-driven neuronal activity is not only mandatory for proper formation of neuronal circuitry, but in addition is required for proper orientation of astrocyte networks in the LSO.

## 2. Results

### 2.1. Expression of Cx43 and Cx30 in the Auditory Brainstem

**Figure 1:**
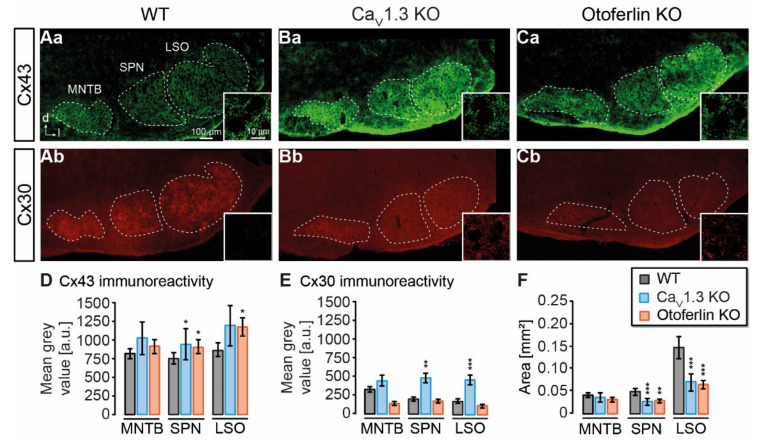
Expression of astrocyte-related connexins in the superior olivary complex (SOC). (**A**–**C**), widefield images showing immunoreactivity of Cx43 and Cx30 in the mouse SOC from the wild type (WT) (**Aa** (Cx43), **Ab** (Cx30)), CaV1.3 knock-out (KO) (**Ba** (Cx43), **Bb** (Cx30)) and otoferlin KO (**Ca** (Cx43), **Cb** (Cx30)). Regions used for mean grey value und area analysis are indicated with dashed lines. Insets: Close ups showing the punctate Cx labeling in the lateral superior olive (LSO) center. (**D**), mean grey values of Cx43 immunofluorescence. Cx43 levels were increased in the SPN from both KO models and in the LSO from otoferlin KO. (**E**), mean grey values of Cx30 immunofluorescence. Cx30 was elevated in the SPN and LSO of Ca_V_1.3 KO. (**F**), area of nuclei in the SOC. SPN and LSO from both KO models exhibited a reduced area compared to the WT. Mean grey values were background subtracted. (**D**–**F**) show mean ± SD. Significance levels in panels (**D**–**F**) were Šidák corrected for two comparisons. The sample size is given in the text of Section 4.2. * *p* < 0.025, ** *p* < 0.005, *** *p* < 0.0005.

In Ca_V_1.3 KO and otoferlin KO mice, neuronal circuitry and nuclei topography are altered in the auditory brainstem [26,35,36]. To assess putative changes in astrocytic coupling, we first analyzed the expression of Cx43 and Cx30 in the SOC containing the MNTB, superior paraolivary nucleus (SPN), and the LSO. As observed before, immunohistochemistry directed against Cx43 and Cx30 resulted in punctate labeling of auditory brainstem nuclei, whereas Cx levels outside of the nuclei, e.g. in the internuclear space, were low (Figure 1Aa–Cb; [1]). Compared to the WT, expression of Cx43 was increased in the SPN from both KO models (Ca_V_1.3 KO: *p* = 0.020; otoferlin KO: *p* = 0.010) and in the LSO from otoferlin KO (*p* = 0.006; Figure 1D). Cx30 levels were elevated in the SPN (*p* = 0.001) and LSO (*p* < 0.001) from Ca_V_1.3 KO, but not from the otoferlin KO (Figure 1E). Cx43 and Cx30 levels were not significantly altered in MNTB from either KO model as compared to the WT (Figure 1D–E).

It was previously shown that deprivation of spontaneous cochlea-driven neuronal activity alters nuclei topography [35]. Thus, we analyzed the size of nuclei and found a 50% smaller coronal nucleus area for the SPN and LSO in both KO models (*p* < 0.001 for all comparisons), however, the coronal MNTB area was not altered (Figure 1F). Notably, the LSO in the Ca_V_1.3 KO lost its typical kidney-like shape (Figure 1B; [35]), whereas this topography was maintained in the otoferlin KO (Figure 1C). Thus, our initial results indicated that astrocytic GJ coupling might be altered due to altered Cx expression and nucleus size.

### 2.2. Electrophysiological Properties of LSO Astrocytes

**Figure 2:**
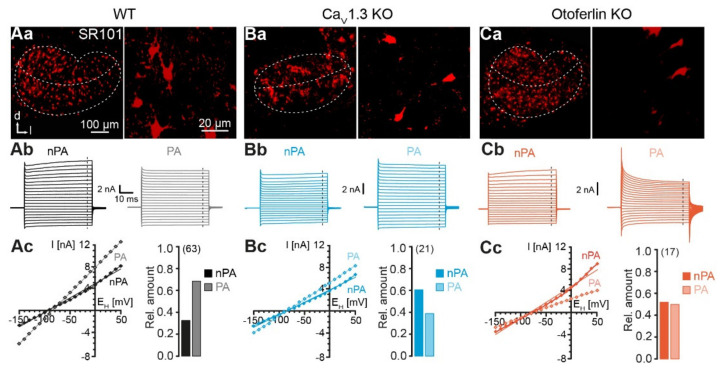
Identification and characterization of LSO astrocytes. (**A**–**C**), identification of astrocytes in the LSO. Confocal images of SR101-labeled astrocytes in the LSO (**left**). SR101-labeling was independent from genetic modification of mouse strains (**Aa**,**Ba**,**Ca**). The border of the LSO and the tonotopic axis are highlighted with dashed lines. In the WT and otoferlin KO mice, the LSO displayed the typical kidney-like shape (**Aa**,**Ca**). In Cav1.3 KO mice, the LSO was elliptic (**Ba**). Higher magnification of SR101-labeled astrocytes in the center of the LSO (**right**). Electrophysiological characterization of astrocytes (**Ab**–**Cc**). Astrocytes were recorded in voltage-clamp mode and step-wise hyper- and depolarized. Non-passive astrocytes (nPA) expressed time- and voltage-dependent outward currents (**left**). Passive astrocytes (PA) exhibited only ohmic currents (**right**) (**Ab**,**Bb**,**Cb**). Current-voltage (*I/V*) relationship was determined at the end of the voltage steps (dashed lines in **Ab**,**Bb**,**Cb**). Due to the presence of outward currents, nPAs and PAs exhibited non-linear and linear *I/V* relationships, respectively (**left**) (**Ac**,**Bc**,**Cc**). Relative amount of n/PAs (**right**). The number of analyzed cells is given in parentheses. The WT data (**Aa**–**Ac**) were part of [9]. Panels Aa left, Ab right, and parts of Ac left were reused from that publication.

To investigate the effect of reduced spontaneous neuronal activity on astrocytic GJ coupling in the auditory brainstem, we chose the LSO as a model region. In previous studies we could show that LSO astrocyte networks are predominantly anisotropic and oriented orthogonally to the tonotopic axis [1,9]. LSO astrocytes were a priori identified using sulforhodamine (SR) 101-labeling [37]. In the WT and both KO models, astrocytes were brightly labeled and were more numerous within the LSO as compared to the area around the nucleus. Analogous to the results from the immunohistochemistry experiment, the astrocyte distribution reflected the typical kidney-like shape of the LSO from the WT and otoferlin KO, and in the Ca_V_1.3 KO astrocytes occupied an elliptic area (Figure 2Aa,Ba,Ca). As described above, the LSO size was reduced in both KO models. In the WT, astrocytes in the LSO center preferentially exhibited a dorsoventral orientation, which is roughly orthogonal to the tonotopic axis (Figure 2Aa; [1]). In contrast, astrocytes in the LSO center from both KO models appeared to be oriented in mediolateral direction, which approximately reflects the tonotopic axis (Figure 2Ba,Ca).

Neuronal circuitry in both KO models shows impaired development, but it was unknown, if the loss of spontaneous cochlea-driven neuronal activity interferes with astrocyte development. We patch-clamped individual LSO astrocytes and characterized their basic electrophysiological properties. Astrocytes from the WT exhibited a highly negative membrane potential (−82.9 ± 4.0 mV, *n* = 63) and a very low membrane resistance (3.7 ± 2.5 MΩ, *n* = 63), which is typical for LSO astrocytes [1,37]. LSO astrocytes from both KO models did not differ in their membrane potential (Ca_V_1.3 KO: −83.2 ± 4.8 mV, *n* = 21, *p* = 0.814; otoferlin KO: −79.0 ± 8.1 mV, *n* = 17, *p* = 0.413) or membrane resistance (Ca_V_1.3 KO: 3.5 ± 1.5 MΩ, *n* = 21, *p* = 0.833; otoferlin KO: 3.7 ± 1.7 MΩ, *n* = 17, *p* = 0.991).

We next hyper- and depolarized astrocytes from the WT and the two KO models to analyze the expression of inward and outward currents (Figure 2Ab,Bb,Cb). According to their elicited current traces, astrocytes could be classified as non-passive astrocytes (nPAs) and passive astrocytes (PAs). Astrocytes mainly showing voltage-activated outward currents resulting in a non-linear current-voltage (*I*/*V*) relationship were designated as nPAs. In turn, astrocytes that primarily expressed ohmic currents and hence displayed a preferentially linear *I/V* relationship, represented PAs. Both astrocytes subtypes are present in the WT and both KO models (Figure 2Ab,Cb). In the WT, most astrocytes exhibited a non-passive phenotype (nPA/PA: 32%/68%, *n* = 63; Figure 2Ac). Interestingly, the relative proportion shifted from PAs towards nPAs in both KO models. In Ca_V_1.3 KO, there are more nPAs than PAs (62%/38%, *n* = 21, *p* < 0.001, Χ^2^ test; Figure 2Bc). In the otoferlin KO, there is an almost equal amount of nPAs and PAs (nPA/PA: 53%/47%, *n* = 17, *p* < 0.001, Χ^2^ test; Figure 2Cc). Thus, our data indicate that astrocytes in KO models do not undergo the normal postnatal transition from nPAs, expressing voltage-activated K^+^ channels, towards PAs, predominantly expressing inwardly rectifying and leak K^+^ channels, and thus partially maintain a phenotype characteristic of an earlier developmental stage [38,39].

### 2.3. Unaltered LSO Astrocyte Network Properties

**Figure 3:**
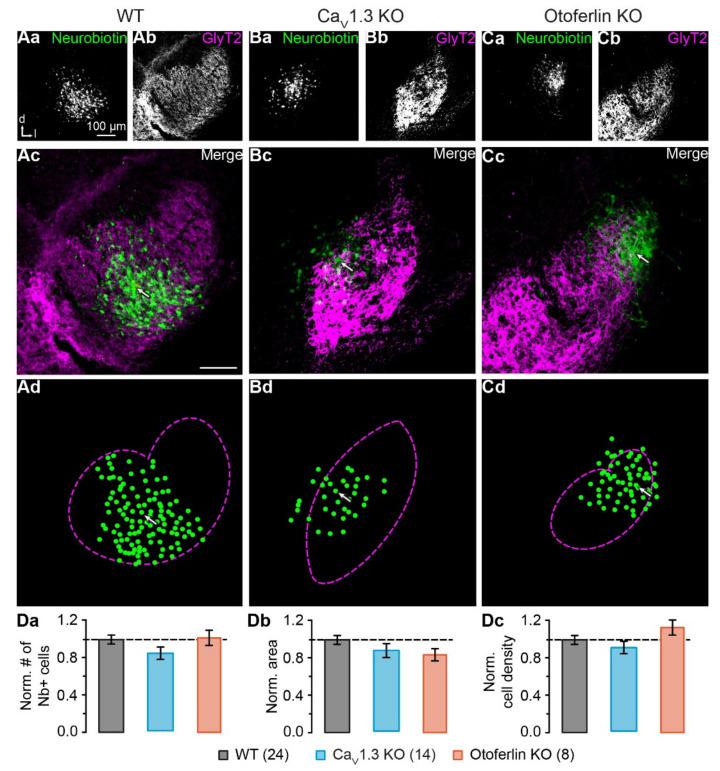
Reconstruction of LSO astrocyte networks. (**A**–**C**), tracer-coupled networks of the WT, Ca_V_1.3 KO, and otoferlin KO mice. The tracer neurobiotin diffused from the patch-clamped astrocyte into coupled cells (**Aa**–**Ac**,**Ba**–**Bc**,**Ca**–**Cc**). Immunohistochemical labeling for glycine transporter (GlyT) 2 highlighted the LSO (**Ab**,**Bb**,**Cb**) and allowed the localization of the network within the nucleus (**Ac**,**Bb**,**Cc**). Cells with fluorescence intensity of at least 1.75-fold background intensity were transferred to a schematized representation and are displayed by green dots (**Ad**,**Bb**,**Cd**). The dotted magenta lines indicate the LSO border as derived from GlyT2 labeling (**Ab**,**Bb**,**Cb**). The arrows in (**Ac**,**Ad**,**Bc**,**Bd**,**Cc**,**Cd**) mark the patched cell. (**D**)**,** network properties. Values were normalized to the WT data, indicated with the dashed line. There were no differences between the number of coupled cells (**Da**), network area (**Db**), or density of coupled cells (**Dc**). The WT data (**Aa**–**Ad**) were part of [9]. Panel (**Aa**–**Ac**) was reused from that publication. (**Da**–**Dc**) show mean ± SD. Number of slices is given in parentheses.

Astrocyte coupling increases during postnatal development, which results in larger networks containing more cells [40,41]. As the percentage of astrocytes that maintained an electrophysiologically earlier developmental phenotype (nPAs) was increased in KO models, we next investigated, if coupling of LSO astrocytes was altered, too. During whole-cell recording the patch-clamped astrocytes were loaded with GJ-permeable tracer neurobiotin. Subsequent tracer visualization revealed labeling of coupled cells, whose brightness declined exponentially with increased distance to the patched cell (Figure 3Aa–Cc). Notably, the LSO borders did not restrict the tracer diffusion.

The semi-automated intensity-based cell detection [9] showed that networks did not differ significantly in basic properties between WT and KO models, i.e. cell number (WT: 64 ± 15, *n* = 24; Ca_V_1.3 KO: 54 ± 12, *n* = 14, *p* = 0.468; otoferlin KO: 65 ± 14, *n* = 8, *p* = 0.129; Figure 3Da), area (WT: 0.043 ± 0.009 mm^2^, *n* = 24; Ca_V_1.3 KO: 0.039 ± 0.007 mm^2^, *n* = 14, *p* = 0.513; otoferlin KO: 0.036 ± 0.007 mm^2^, *n* = 8, *p* = 0.644; Figure 3Db), and cell density (WT: 1484 ± 331 cells/mm^2^, *n* = 24; Ca_V_1.3 KO: 1398 ± 331 cells/mm^2^, *n* = 14, *p* = 0.493; otoferlin KO: 1816 ± 384 cells/mm^2^, *n* = 8, *p* = 0.137; Figure 3Dc). Thus, in contrast to the electrophysiological phenotype, the network size in both KO models was unchanged and did thereby not reflect the properties of an earlier developmental stage.

### 2.4. Disturbed LSO Astrocyte Network Topography

**Figure 4:**
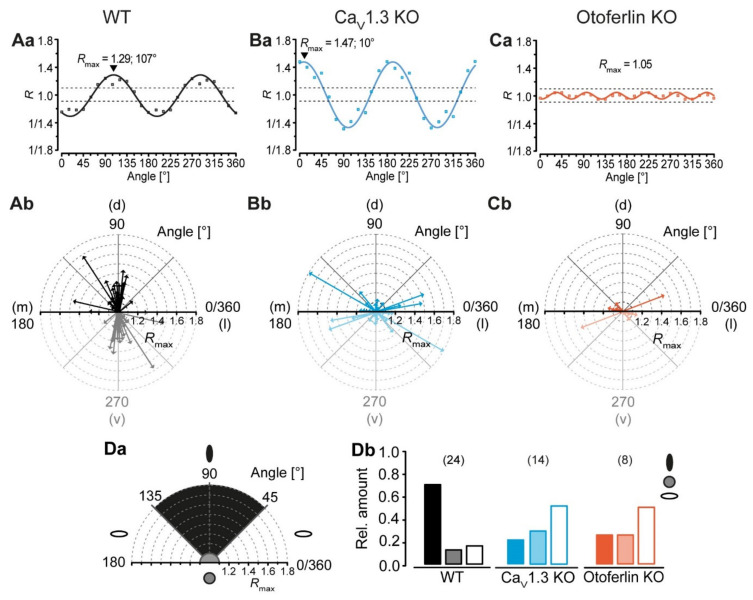
LSO astrocyte networks show a disturbed topography in Ca_V_1.3 KO and otoferlin KO mice. (**A**–**C**), analysis of network topography. The coordinate system was step wise rotated and the ratio of tracer extent was calculated using the vector means approach. The anisotropy and orientation of networks in the center of the LSO were determined using a sinusoidal function (**Aa**–**Ca**). Shown, are representative sinusoidal fits of the anisotropic networks that were oriented orthogonally (WT; Aa) and parallel to the tonotopic axis (Ca_V_1.3 KO; Ba) as well as a fit of an isotropic network with no preferential orientation (otoferlin KO; Ca). (**Aa**–**Ca**) refer to networks shown in Figure 3Aa–Cd. Radar diagrams displaying the anisotropy (*R_max_* > 1.1: anisotropic; *R_max_* ≤ 1.1: isotropic) and orientation *α* of tracer-coupled networks (**Ab**–**Cb**). The mediolateral (m-l) axis resembles the tonotopic axis, the dorsoventral (d–v) axis resembles the orientation of isofrequency bands, which are oriented orthogonal to the tonotopic axis. In WT mice, most tracer-coupled networks showed a preferential orientation orthogonal to the tonotopic axis (d–v; 45 ≤ *α* < 135°) (**Ab**). In contrast, the majority of anisotropic astrocyte networks in Ca_V_1.3 KOs and otoferlin KOs were aligned parallel to the tonotopic axis (m-l; *α* < 45° and *α* ≥ 135°) (**Bb**–**Cb**). (**D**), classification of networks. Astrocyte networks were affiliated to three classes by their shape *R_max_* and orientation *α* (class 1, black ellipse: anisotropic and oriented orthogonally to tonotopic axis; class 2, grey circle: isotropic; class 3: anisotropic and oriented parallel to the tonotopic axis; **Da**). In WT mice, most LSO astrocyte networks were categorized into class 1. In contrast, tracer-coupled networks in KOs were predominantly affiliated to class 3 (**Db**). The WT data (**Aa**–**Ab**) were already part of the following study: [9]. Panel Ab was reused from that publication.

LSO astrocyte networks are predominantly orthogonal to the tonotopic axis [1,9]. We next analyzed, if this preferential orientation is maintained in KO models. Network anisotropy was analyzed using our vector-based approach with subsequent meta-analysis [9]. Therefore, we applied a sinusoidal fit to the data to calculate the shape (*R_max_*) and orientation (*α*) relative to the dorsoventral axis (Figure 4Aa,Ba,Ca). In case of anisotropic tracer-coupled networks comprising two axes of symmetry, rotating the coordinate system resulted in a ratio that oscillates two times per full turn (Figure 4Aa,Ba). In contrast, spherical networks with more than two axes of symmetry oscillated with a considerably higher frequency (Figure 4Ca).

**Figure 5:**
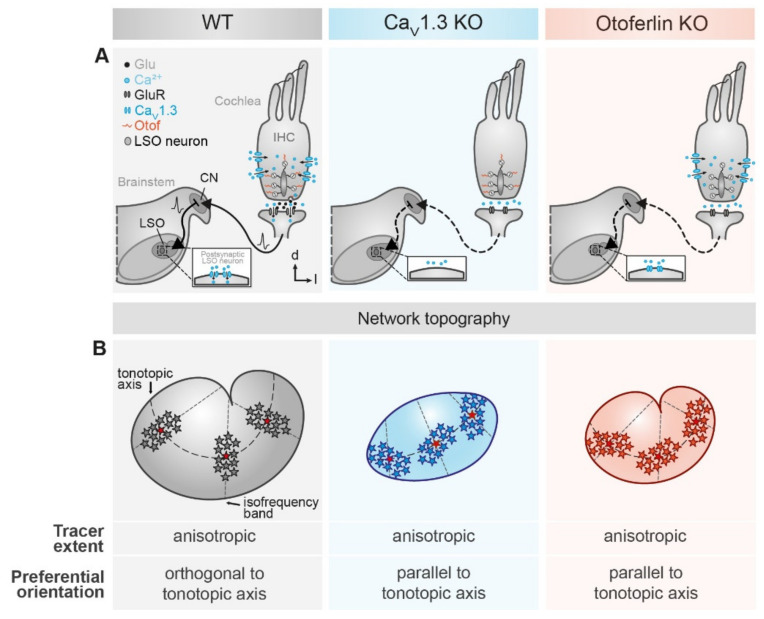
Summary of LSO astrocyte network topography in mouse models for human hereditary deafness. (**A**) schematic drawings depicting the subcellular modifications of the different mouse models. Compared to the WT (**left**), absence of Ca_V_1.3 (**middle**) and otoferlin (**right**) from cochlear inner hair cells prevents Ca^2+^ entry into the inner hair cell and Ca^2+^ detection, respectively. Subsequently, exocytotic glutamate release is inhibited. Thereby, spontaneous activity of inner hair cells does not result in vesicle fusion, synaptic transmission, and subsequent activation of the auditory pathway. (**B**) main result of network analysis. LSO astrocyte networks are preferentially anisotropic and oriented orthogonally to the tonotopic axis (**left**). By contrast, networks in Ca_V_1.3 KO (**middle**) and otoferlin KO mice (**right**) are predominantly oriented parallel to the tonotopic axis. Furthermore, the area of the LSO is reduced in both KO models. Moreover, the kidney-like shape of the LSO is lost in the Ca_V_1.3 KO.

As expected (cf. [1]), the WT LSO astrocyte networks were predominantly anisotropic and oriented orthogonally to the tonotopic axis (71%, 17/24; Figure 4Ab,Db). Only 13% (3/24) of the WT tracer-coupled networks were spherical and 17% (4/24) were anisotropic with a preferential orientation parallel to the tonotopic axis (Figure 4Ab,Db). Similar to this, most LSO astrocyte networks in Ca_V_1.3 KO and otoferlin KO mice were anisotropic (Figure 4Bb,Cb). However, tracer-coupled networks in both KO models showed a different predominant orientation. Half of the networks in Ca_V_1.3 KO (7/14) and otoferlin KO mice (4/8) were oriented parallel to the tonotopic axis (Figure 4Bb,Cb,Db). Less were spherical (Ca_V_1.3: 29%, 4/14; Figure 4Bb,Db; Otof: 25%, 2/8; Figure 4Cb,Db) or oriented orthogonally to the tonotopic axis (Ca_V_1.3 KO: 21%, 3/14; Figure 4Bb,Db; otoferlin KO: 25%, 2/8; Figure 4Cb,Db). Thus, the predominant direction of tracer spread and, accordingly, the preferred orientation of LSO astrocytes networks turned in both KO models by 90°, as compared to the WT. As a consequence, there might be increased gap junction-related cross-talk along the tonotopic axis (Figure 5).

Taken together, our results show that deprivation of spontaneous cochlea-driven neuronal activity does not per se distort astrocyte coupling in the LSO. However, LSO astrocyte network orientation is largely altered.

## 3. Discussion

In the present study, we have investigated the influence of absent spontaneous cochlea-driven neuronal activity on gap junctional tracer coupling in LSO astrocytes. To do so, we used two mouse models of hereditary deafness—a Ca_V_1.3 KO and an otoferlin KO. As we have previously demonstrated a strong anatomo-functional correlation between neuronal circuitry and glial GJ network topography, we hypothesized that the altered neuronal circuitry in these two mouse models is also reflected in altered astrocytic networks. Our data show that the expression of astrocytic Cx is partially increased in the LSO, but the extent of tracer coupling is not altered. Most GJ networks are still anisotropic, but are oriented along the tonotopic axis in the KOs, thus correlating with the disturbed neuronal circuitry.

### 3.1. Connexin Expression in KO Models

GJ coupling depends on Cx expression levels. In the barrel cortex, high Cx expression within barrels correlates with strong GJ coupling, whereas lower Cx levels in the septa between the barrels result in weaker coupling [7,42]. Furthermore, Cx expression is upregulated during development [1,2,43,44], which increases GJ coupling [40,41]. Accordingly, loss of spontaneous cochlea-driven neuronal activity leading to impaired developmental maturation of neuronal circuitry [25,26,35,36] might have kept Cx expression in the LSO at an earlier developmental state as well. Here, we even found moderately increased Cx levels in the LSO, while the nucleus area was reduced in Ca_V_1.3 KO and otoferlin KO mice (Figure 1; [35]). However, neither the increase in Cx levels nor the reduced nucleus size affected the size of tracer-coupled networks (Figure 3).

### 3.2. Activity-Dependent Alteration of Astrocyte Network Topography

LSO astrocytes in the two KO models exhibited similar basic membrane properties as reported earlier for the WT, namely a very negative membrane potential and a low membrane resistance [1,37]. Thus, expression of Kir and K_2_P channels, which set both membrane potential and membrane resistance [45], is independent from spontaneous cochlea-driven neuronal activity. However, LSO astrocytes in both KO models exhibited more often a non-linear *I*/*V* relationship (Figure 2), which is indicative of partially impaired development, as they stayed in an earlier developmental state (cf. [38,39]). There was no significant alteration of network size, which is a bit surprising as Cx levels were moderately increased. Moreover, most tracer-coupled networks were anisotropic in both KO models (Figure 4). However, the disturbed refinement of neuronal circuitry is paralleled by an altered network orientation. Whereas networks in the WT were predominantly oriented orthogonally to the tonotopic axis [1,9], networks in KO models were predominantly oriented parallel to the tonotopic axis (Figure 4). Thus, spontaneous cochlea-driven neuronal activity per se is not required for the formation of anisotropic LSO astrocyte networks. However, it drives astrocytes and networks to be predominantly oriented orthogonally to the tonotopic axis.

### 3.3. Mechanism Underlying the Altered Network Topography

There must be at least two mechanisms in the LSO directing, on the one hand, network anisotropy and on the other hand, network orientation. In the two KO models used, loss of spontaneous cochlea-driven neuronal activity only interferes with network orientation, but not with network anisotropy per se (Figure 4). Anisotropic tracer coupling is present in different brain regions and can have different origins. In the barrel cortex and barreloid thalamus, anisotropy of glial GJ networks arises from restricted coupling across the barrels [7,8]. So far, such restrictions were not found in the LSO, since GJ networks cross nuclear borders [1]. In contrast, network anisotropy in the hippocampus and in the LSO originates from anisotropic topography of astrocyte processes [1,9,46,47]. The astrocyte polarization in the hippocampus depends on a non-channel function of Cx30 [46]. However, polarization of astrocytes and subsequent orientation of GJ networks in the LSO must be independent from Cx30 as it is virtually absent at the early postnatal stage (P10–12) investigated in this study [1]. Moreover, it is rather unlikely that the slightly elevated Cx30 expression in the Ca_V_1.3 KO interferes with GJ network orientation, as there is no elevation of Cx30 expression in the otoferlin KO (Figure 1) and both KO models show the same alteration of GJ network orientation (Figure 4).

Astrocyte morphology correlates with topography of GJ networks in the auditory brainstem [1,2]. The changed orientation of astrocyte processes in the LSO in both KO models is likely to be responsible for the alteration of preferred GJ network orientation (Figure 2 and Figure 4). However, the following question needs to be answered: What is the link between the lack of spontaneous cochlea-driven neuronal activity and alteration of astrocyte and GJ network topography?

### 3.4. Signaling between Astrocytes and Neurons

Spontaneous cochlea-driven neuronal activity is not only important for postnatal refinement of neuronal circuitry and dendrite topography [26,35,36], but is also required for the formation of GJ networks that are oriented predominantly orthogonally to the tonotopic axis. However, the interplay between astrocytes and neurons during this early postnatal phase is not clear as we do not know who signalizes whom to mature. There are basically two opposing possibilities: (1) Astrocyte topography and subsequent GJ network orientation precede and induce neuronal refinement, or (2) neuronal circuitry directs astrocytes and subsequently GJ networks to arrange properly. Another aspect is the question—until which point do the astrocyte and network maturation processes require cochlea-driven neuronal activity? This question can be addressed in future studies using, for example, the Pou4f3^DTR^ mouse line, in which inner hair cells can be ablated by injection of diphtheria toxin [48].

In the avian auditory brainstem astrocyte-secreted factors are required to modulate dendrite topography and synapse distribution [49,50]. Therefore, the absence of spontaneous cochlea-driven neuronal activity likely does not induce neuronal refinement directly, but requires astrocyte–neuron signaling. However, GJ networks are affected themselves. This suggests that there must be in addition a communication between neurons and astrocytes, whose absence renders GJ network orientation in the KO models. This idea is further supported by the fact that the knocked-out targets, namely Ca_V_1.3 and otoferlin, are localized in neurons and inner hair cells, respectively, but not in astrocytes [51,52]. In contrast, the still maintained preferred anisotropic topography of GJ networks indicates that this is an intrinsic property of LSO astrocytes and is independent from spontaneous cochlea-driven neuronal activity.

### 3.5. Conclusion

Taken together, our results demonstrate that spontaneous cochlea-driven neuronal activity is not exclusively mandatory for the proper formation of neuronal circuitry, but in addition, is crucial for the proper formation of GJ networks. Hence, GJ network topography reflects disturbed neuronal topography in the investigated mouse models. The signaling path between astrocytes and neurons has to be further analyzed.

## 4. Materials and Methods

Experiments were performed on WT C57BL/6 mice, Ca_V_1.3 KO mice [33] and otoferlin KO mice [34] of both genders at postnatal days 10–12 in accordance with the German law for conducting animal experiments. Animals were bred at a 12 h day/night cycle and received food and water ad libitum. Breeding was approved by the regional council of Rhineland-Palatinate (23 177-07/G 15-2-076; 24 August 2016). In accordance with the German animal welfare act (TSchG), no additional approval for post mortem removal of brain tissue was necessary. All chemicals were purchased from Sigma-Aldrich (St. Louis, MO, USA) or AppliChem (Darmstadt, Germany), if not stated otherwise.

### 4.1. Genotyping

At 3–5 days after birth and directly after preparation of brain tissue, a tail biopsy was taken. First, biopsies were digested in 200 μL 25 mM NaOH and 0.2 mM ethylenediaminetetraacetic acid (EDTA) for 1 h at 95° Celcius (C) at 300 rpm in a twitter (Thriller, Peqlab, VWR, Darmstadt, Germany) to isolate the DNA. Afterwards, 200 μL 40 mM tris(hydroxymethyl)aminomethane (Tris)—HCl, pH 5, was added to neutralize the solution and products were centrifuged for 9–10 min at 15–20 °C at 13,000 rpm (Biofuge fresco, Heraeus, Thermo Fisher Scientific, Waltham, MA, USA). For the following polymerase chain reaction (PCR), 200 μL of the supernatant was decanted, since this contained the DNA. The PCR solution contained the master mix (Table 1) as well as the decanted supernatant. PCR protocols were performed as listed in Table 1. For otoferlin PCR, a restriction enzyme was used to determine genotypes. Therefore, a second digestion was performed with a solution containing 3 μL autoclaved H_2_O, 1 μL 10× NEB 3 enzyme buffer and 1 μL BGI II-enzyme (Biolabs, Frankfurt am Main, Germany) times the samples plus 5 μL of the PCR product.

Next, visualization of the DNA bands in the gel was achieved by adding 4 μL sample buffer (40 mM Tris, 20 mM acetic acid, 1 mM EDTA with 40% glycerol and Xylene cyanol). Then, 5 μL DNA ladder (Hyperladder Bio-33040, Bioline, Meridian Biosciences, Memphis, TN, USA) was loaded into the first lane of each 1.5% agarose gel (1.5% agarose and 0.001% EtBr diluted in tris-acetate-EDTA buffer). The other lanes were filled with 14 μL of each probe and were run for 30–35 min at 90–95 V. To develop the gel and visualize the bands, gels were put into a chamber (Biometra Tl1, LTF Labortechnik, Wasserburg, Germany).

### 4.2. Immunohistochemistry

Animal perfusion and tissue preparations were performed as described earlier [1]. The tissue was subsequently processed for Cx43 and Cx30 antibody labeling. Tissues were transferred to phosphate buffered saline (PBS) and cut into 25–30 μm thick slices using a microtome (HM650V, Microtome, Microm International GmbH, Thermo Fisher Scientific, Waltham, MA, USA). Slices were mounted on glass slides (SuperFrost Plus, VWR, Darmstadt, Germany) for on-slide labeling. Unspecific binding sites were blocked with 0.25% triton X-100 and 2% normal goat serum (NGS; Gibco, Thermo Fisher Scientific, Waltham, MA, USA) for 1 h at room temperature (RT). Primary antibodies (rabbit anti-connexin 43, C6219, Sigma-Aldrich, St. Louis, MO, USA; rabbit anti-connexin 30, 700258, Invitrogen, Thermo Fisher Scientific, Waltham, MA, USA) were diluted 1:500 in 0.25% triton X-100 and 2% NGS and applied over night at +4 °C. Since both Cx antibodies were raised in the same host species, stainings were performed on separate sets of fixed slices. After washing with 0.25% triton X-100 and 2% NGS, tissue slices were incubated with secondary antibody (Alexa Fluor (AF) 488 goat anti-rabbit, A-11034, Invitrogen, Thermo Fisher Scientific, Waltham, MA, USA) diluted 1:100 in 2% NGS for 70 min at RT. After washing with PBS, slices were provided with coverslips in 10% DABCO (Fluka, Sigma-Aldrich, St. Louis, MO, USA) in MOWIOL (Calbiochem, Merck, Darmstadt, Germany).

Overview images were documented using a motorized upright widefield microscope (Nikon Eclipse 90i: Plan Fluor 10×/0.30, Nikon Instruments, Tokio, Japan) equipped with a DS-Q1Mc camera (Nikon Instruments, Tokio, Japan) and a FITC filter set (EX: 465–495 nm; DM: 505 nm; BA: 515–555 nm). All settings were kept constant when comparing immunolabeled areas and stainings. High-resolution images showing the center of auditory brainstem nuclei were taken on a motorized confocal laser scanning microscope (Nikon Eclipse C1 mounted at an E600FN: Plan Apo VC 60x/1.40 Oil, Nikon Instruments, Tokio, Japan). Fluorophores were detected with an Argon laser (excitation: 488 nm; emission collected at >515 nm; Melles Griot, Bensheim, Germany) in combination with EZ-C1 3.91 Silver Version software (Nikon Instruments, Tokio, Japan). A minimum of 3 slices were analyzed per nucleus and genotype: WT (Cx43/30): *n* = 3–5/3–8; Ca_V_1.3 KO: *n* = 19–23/11–12; otoferlin KO: *n* = 5/6–8. The number of slices used for the analysis of nucleus area is the cumulated number of slices used for Cx43 and Cx30 for each nucleus and genotype. Selection and documentation of slices was done blind. For background correction of signal intensities, negative controls were performed and resulting mean background levels for each nucleus were subtracted.

### 4.3. Preparation of Acute Tissue Slices

Acute coronal brainstem slices were prepared as described earlier [37]. In brief, brains were quickly dissected after decapitation and transferred into ice-cold cutting solution containing (in mM): 26 NaHCO_3_, 1.25 NaH_2_PO_4_, 2.5 KCl, 1 MgCl_2_, 2 CaCl_2_, 260 d-glucose, 2 Na-pyruvate, and 3 myo-inositol, pH 7.4, bubbled with carbogen (95% O_2_, 5% CO_2_). Thereafter, slices were transferred to artificial cerebrospinal fluid (ACSF) containing (in mM): 125 NaCl, 25 NaHCO_3_, 1.25 NaH_2_PO_4_, 2.5 KCl, 1 MgCl_2_, 2 CaCl_2_, 10 d-glucose, 2 Na-pyruvate, 3 myo-inositol, and 0.44 ascorbic acid, pH 7.4, bubbled with carbogen. 270-μm-thick slices were cut using a vibratome (VT1200 S, Leica, Wetzlar, Germany). For a priori identification of astrocytes, slices were incubated for 30 min at 37 °C in 0.5–1 μM SR101 dissolved in ACSF and washed for another 30 min at 37 °C in SR101-free ACSF. Afterwards, slices were kept at RT until experiments were performed.

### 4.4. Electrophysiology and Tracer Loading

Whole-cell patch-clamp experiments were performed at RT with an upright microscope equipped with infrared differential interference contrast (Eclipse FN1, Nikon Instruments, 60× water immersion objective, N.A. 1.0, Tokio, Japan) and an infrared video camera (XC-ST70CE, Hamamatsu, Shizuoka, Japan) using a patch-clamp EPC10 amplifier and “PatchMaster” software (HEKA Elektronik, Lambrecht, Germany). The pipette solution contained (in mM): 140 K-gluconate, 5 EGTA (glycol-bis(2-aminoethylether)-N,N′,N′,N′-tetraacetic acid), 10 HEPES (*N*-(2-hydroxyethyl)piperazine-*N*′-2-ethanesulfonic acid), 1 MgCl_2_, 2 Na_2_ATP, and 0.3 Na_2_GTP, pH 7.3. The pipette solution additionally contained a cocktail of the GJ-impermeable dye AF568 (100 μM, Invitrogen, Thermo Fisher Scientific, Waltham, MA, USA) and the GJ-permeable tracer neurobiotin (1%, Vector Laboratories, Inc., Peterborough, UK) to mark the patched cell and label the coupling network, respectively [1,2]. Patch pipettes were pulled from borosilicate glass capillaries (GB150(F)-8P, Science Products, Hofheim am Taunus, Germany) using a horizontal puller (P-87, Sutter Instruments, Novato, CA, USA) and had a resistance of 2–8 MΩ.

Astrocytes were patched in the central part of the LSO, where the mediolateral and dorsoventral axes are roughly tangential and orthogonal to the tonotopic axis, respectively. Astrocytes were recorded in voltage-clamp mode and held at −85 mV, which is close to their resting membrane potential [1,37]. The (fast) pipette capacitance was compensated. In standard whole-cell configuration the total input resistance (*R*_In_) consists of membrane resistance (*R*_M_) and series resistance (*R*_S_) that are arranged in series [53]. They were calculated from currents recorded during hyperpolarizing voltage steps (Δ*U* = 5 mV). *R*_In_ is given by (Equation (1)):

(1) $$R_{\mathrm{In}}=\frac{U_{2}-U_{1}}{I_{2}-I_{1}}$$

with *U*_1_ is −85 mV, *U*_2_ is −90 mV. *I*_1_ and *I*_2_ are the recorded steady-state currents at *U*_1_ and *U*_2_, respectively. *R*_S_ was calculated by (Equation (2)):

(2) $$R_{S}=\frac{U_{2}-U_{1}}{I_{\mathrm{peak}}-I_{1}}$$

with *U*_1_, *U*_2_, and *I*_2_ are the same parameters as given in Equation (1) and *I*_peak_ is the maximal current at the initial phase when clamping from *U*_1_ to *U*_2_. Finally, *R*_M_ was calculated by (Equation (3); [54]):

(3) $$R_{M}=R_{\mathrm{In}}-R_{S}$$

Measurements were rejected if the *R*_S_ exceeded 15 MΩ to ensure sufficient electrical and diffusional access to the patched cell [55]. The liquid junction potential was not corrected. Astrocytes were characterized by applying a standard step protocol ranging from −150 mV to +50 mV with 10 mV increments and step duration of 50 ms to determine their *I/V* relationship. The resulting current traces were sampled at 50 kHz and online filtered at 2.9 kHz. Data were analyzed using “IGOR Pro” software (WaveMetrics, Lake Oswego, OR, USA).

### 4.5. Visualization of Coupled Cells

GJ networks and nucleus boundaries were visualized as described earlier [1,9]. Fixed slices were processed at RT. First, slices were washed three times in PBS (containing NaCl, Na_2_HPO_4_*2H_2_O, NaH_2_PO_4_*H_2_O; pH 7.4). Membrane permeabilization was achieved by incubation in 0.25% triton X-100 for 30 min. Thereafter, slices were washed again in PBS. Neurobiotin was identified by incubating slices for 3 h with avidin AF488 (50 μg/mL, Invitrogen, Thermo Fisher Scientific, Waltham, MA, USA) and slices were washed again. Since tracer coupling differs within and dorsal to the LSO [1], glycine transporter (GlyT) 2-staining was used to identify the relative position of the patched cell and the network within the LSO. Avidin-labeled slices were again permeabilized for 30 min in 0.25% triton X-100. Unspecific binding sites were blocked for 1 h in a solution containing 2% bovine serum albumin (BSA), 11.1% NGS (PAA laboratories, Cölbe, Germany), and 0.3% triton X-100. The slices were then incubated overnight (about 20 h) at +4 °C with primary antibody (rabbit anti-GlyT2, AB1773, Millipore, Burlington, MA, USA) diluted 1:2000 in 1% BSA, 1% NGS, and 0.3% triton X-100. The next steps were performed at RT. After washing in PBS, slices were incubated for 90 min with the secondary antibody (goat anti-rabbit AF647, A-21450, Invitrogen, Thermo Fisher Scientific, Waltham, MA, USA) diluted 1:300 in 1% BSA, 1% NGS, and 0.3% triton X-100. Finally, slices were washed in PBS and mounted in 2.5% Dabco on glass slides.

SR101-labeling, network tracing and immunohistochemical stainings were documented at a confocal microscope (Zeiss LSM700: EC Plan-Fluor 10x/0.3; Plan-Apochromat 63x/1.4 Oil) in combination with ZEN software (Zeiss, Oberkochen, Germany), respectively. Fluorophores were detected as follows (excitation wavelength/filtered emission wavelength): AF488 (488 nm/505–530 nm), AF568 (543 nm/>560 nm), AF647 (639 nm/>640 nm), and SR101 (561 nm/580–620 nm). To improve the quality of confocal micrographs and reduce background fluorescence, a Kalman filter was used (averaging of four identical image sections). Images were processed using “FIJI” software [56].

### 4.6. Analysis of Network Topography

To avoid unconscious experimenter-based corruption of data, coupled cells were identified using an intensity-based detection method [9]. Only cells surpassing a threshold of 1.75 times background intensity were incorporated in the analysis (Figure 3Ad,Bb,Cd). Subsequent vector-based calculation of network topography was used for an automated analysis [9]. Here, the network was divided into four 90° sectors and the sum vector for each sector was calculated. The length of these vectors was normalized to the number of cells in each sector. *R* is the quotient of the normalized *y* value and the normalized *x* value (Equation (4)):

(4) $$R=\frac{\frac{\left|\vec{y_{1A}}\right|}{n_{1A}}+\frac{\left|\vec{y_{1B}}\right|}{n_{1B}}}{\frac{\left|\vec{x_{2A}}\right|}{n_{2A}}+\frac{\left|\vec{x_{2B}}\right|}{n_{2B}}}$$

where $\left|\vec{y_{1A}}\right|$, $\left|\vec{y_{1B}}\right|$, $\left|\vec{x_{2A}}\right|$, $\left|\vec{x_{2B}}\right|$ are the absolute values of the sum vectors of the sectors 1A, 1B, 2A, and 2B, respectively, and *n_1A_*, *n_1B_*, *n_2A_* and *n_2B_* are the number of cells in respective sectors. Then, the coordinate system was rotated and the ratio was recalculated in 15° steps. A sinusoidal function (Figure 4Aa,Ba,Ca; Equation (5)) was fitted to the data:

(5) $$R=A_{0}+A\sin(\omega \alpha +(\varphi +\frac{3}{4}\pi ))$$

where $A_{0}$ is the offset, ω is the circular frequency, α is the angle and φ is the phase shift. The highest Ratio (*R_max_ = A_0_ + A*) of the fit reveals the angle of maximal anisotropy of a single network. Therefore, networks were classified into three groups depending on *R_max_* and their preferential orientation *α* (Figure 4Da): (1) *R_max_* > 1.1 and 45° < *α* ≤ 135°, anisotropic and orthogonal to the tonotopic axis, (2) *R_max_* ≤ 1.1, round and (3) *R_max_* > 1.1 and 0° ≤ *α* < 45° or 135° < *α* ≤ 180°, anisotropic and parallel to the tonotopic axis.

### 4.7. Statistics

Results are provided as mean ± SD. Data were statistically analyzed using WinSTAT (R. Fitch Software, Bad Krozingen, Germany) and tested for normal distribution with the Kolmogorov–Smirnov test. In case of normal distribution, results were assessed by two-tailed, unpaired Student’s *t*-tests. Otherwise, results were assessed by a Mann–Whitney *U*-test. Differences in distribution of classes were analyzed between the WT and the two mouse models using a Χ^2^ test. *p* represents the error probability, * *p* < 0.05, ** *p* < 0.01, *** *p* < 0.001. *n* represents the number of recorded cells or analyzed networks (/slices). In case of multiple comparisons data were statistically analyzed by the tests described above under post hoc Šidák correction of critical values [57]: two comparisons: Figure 1D–F and Figure 3D: * *p* < 0.025, ** *p* < 0.005, *** *p* < 0.0005.

### 4.8. Additional Information

The WT data, as well as Figure 2Aa left, Ab right, and parts of Ac, Figure 3Aa–Ac, and Figure 4Ab were taken from [9] in accordance to the terms and conditions of the Creative Commons Attribution (CC BY) license (http://creativecommons.org/licenses/by/4.0/).

## Figures and Tables

**Table 1:** Master mixes for PCR solutions and protocols used for genotyping of WT and KO mice.

Geno-Type	H_2_O	5× PCR Buffer	Forward Primer	Reverse Primer	Taq Poly-Merase	PCR Protocol	Amplicon Size (bp)
WT	7.7 μL	4.0 μL	2.0 μL, 5 pmol/μL, 5′-GCA AAC TAT GCA AGA GGC ACC AGA-3′	2.0 μL, 5 pmol/μL, 5′-TAC TTC CAT TCC ACT ATA CTA ATG CAG GCT-3′	0.3 μL	2 min 92 °C; 20 s 52 °C; 30 s 72 °C; 30 cycles (20 s 92 °C; 20 s 52 °C; 30 s 72 °C); 7 min 72 °C; 15 °C cool down	300
CaV1.3 KO	7.9 μL	4.0 μL	2.0 μL, 5 pmol/μL, 5′-TTC CAT TTG TCA CGT CCT GCA CCA-3′	2.0 μL, 5 pmol/μL, 5′-TAC TTC CAT TCC ACT ATA CTA ATG CAG GCT-3′	0.1 μL	2 min 92 °C; 20 s 52 °C; 30 s 72 °C; 43 cycles (25 s 92 °C; 20 s 52 °C; 30 s 72 °C); 7 min 72 °C; 15 °C cool down	450
Otoferlin KO	7.9 μL	4.0 μL	0.5 μL, 10 pmol/μL, 5′-TAC TGC CCA CAT GAG CTT TG-3′	0.5 μL, 10 pmol/μL, 5′-CAG AGG AAT CCA GCT GAA GG-3′	0.1 μL	2 min 95 °C; 30 s 95 °C; 34 cycles (20 s 57 °C; 30 s 72 °C); 5 min 72 °C; 15 °C cool down	186/163 (WT), 349 (KO)

## Abbreviations

ACSF	Artificial cerebrospinal fluid
AF	Alexa fluor
BSA	Bovine serum albumin
Ca_V_	Voltage-activated calcium channel
Cx	Connexin
EDTA	Ethylenediaminetetraacetic acid
EGTA	Glycol-bis(2 aminoethylether)-N,N′,N′,N′-tetraacetic acid
GJ	Gap junction
GlyT	Glycine transporter
HEPES	N (2 hydroxyethyl)piperazine-N′ 2 ethanesulfonic acid
IC	Inferior colliculus
KO	Knock-out
LSO	Lateral superior olive
MNTB	Medial nucleus of the trapezoid body
NGS	Normal goat serum
nPA	Non-passive astrocyte
PA	Passive astrocyte
PBS	Phosphate buffered solution
PCR	Polymerase chain reaction
*R*	Ratio
RT	Room temperature
SOC	Superior olivary complex
SPN	Superior paraolivery nucleus
SR101	Sulforhodamine 101
Tris	Tris(hydroxymethyl)aminomethane
WT	Wild type
